# Upregulated expression of indoleamine 2, 3-dioxygenase in CHO cells induces apoptosis of competent T cells and increases proportion of Treg cells

**DOI:** 10.1186/1756-9966-30-82

**Published:** 2011-09-14

**Authors:** Jingyan Sun, Jinpu Yu, Hui Li, Lili Yang, Feng Wei, Wenwen Yu, Juntian Liu, Xiubao Ren

**Affiliations:** 1 Department of Breast Oncology, Tianjin Medical University Cancer Institute and Hospital, Tiyuanbei, Huanhuxi Road, Hexi District, Tianjin, 300060, China; 2 Department of Immunology, Key laboratory of Cancer Prevention and Therapy, Tianjin Medical University Cancer Institute and Hospital, Tiyuanbei, Huanhuxi Road, Hexi District, Tianjin, 300060, China

**Keywords:** Indoleamine-Pyrrole 2, 3-Dioxygenase, breast neoplasms, immune tolerance, CHO Cells, regulatory T-Lymphocytes

## Abstract

**Introduction:**

The inflammatory enzyme indoleamine 2, 3-dioxygenase (IDO) participates in immune tolerance and promotes immune escape of IDO+ tumors. A recent hypothesis suggested that IDO may contribute to the differentiation of new T regulatory cells (Tregs) from naive CD4+ T cells. In this study we investigated the role of IDO in induction of immunosuppression in breast cancer by increasing the apoptosis of T cells and the proportion of Tregs.

**Methods:**

An IDO expression plasmid was constructed and Chinese hamster ovary (CHO) cells were stably transfected with human IDO. Purified CD3+ T cells were isolated from the peripheral blood monouclear cells of breast cancer patients. After co-culturing IDO expressing or untransfected (control) CHO cells with T cells, T cells apoptosis were determined by flow cytometry analysis and annexin-V and PI staining. The proportion of the regulatory T cell (Tregs [CD4 + CD25 + CD127-]) subset was measured by flow cytometry analysis. T cells total RNA and cellular protein samples were isolated for detecting Foxp3 gene and protein expression.

**Results:**

IDO transgenic CHO cells yielded high levels of IDO enzymatic activity, resulting in complete depletion of tryptophan from the culture medium. We found that apoptosis occurred in 79.07 ± 8.13% of CD3+T cells after co-cultured with IDO+ CHO cells for 3 days and the proportion of CD4 + CD25 + CD127- T cells increased from 3.43 ± 1.07% to 8.98 ± 1.88% (*P *< 0.05) as well. The specific inhibitor of IDO,1-MT efficiently reversed enhancement of T cells apoptosis and amplification of Tregs in vitro. Increased expression of Foxp3, a key molecular marker of Tregs, was confirmed by RT-PCR, real-time RT-PCR and Western blot analysis at the same time.

**Conclusions:**

These results suggest that IDO helps to create a tolerogenic milieu in breast tumors by directly inducing T cell apoptosis and enhancing Treg-mediated immunosuppression.

## Introduction

The molecular mechanisms underlying tumor-induced tolerance are the subject of active research, and a number of contributing mechanisms have been identified. Indoleamine 2, 3-dioxygenase (IDO/INDO), an important enzyme in the metabolism of tryptophan, catalyzes the rate-limiting step of tryptophan degradation along the kynurenine pathway. Reduction in the local tryptophan concentration and generation of tryptophan metabolites can suppress T cell proliferation or induce T cell apoptosis [1,2], and IDO has been implicated in the endogenous induction of peripheral tolerance and immunosuppression [3,4]. In addition, many human solid tumors express IDO, indicating that it may contribute to the induction of tumor tolerance [5-8].

Regulatory T cells (Tregs [CD4+CD25+CD127-]) can inhibit most types of immune responses and are emerging as a key component of acquired tolerance to tumors [9]. Increased Treg activity facilitates tumor growth, whereas depletion of Tregs allows for effective anti-tumor immune responses [10]. Previous studies have shown that IDO is expressed in tumor-draining lymph nodes. Interestingly, we previously found that IDO expression in primary breast cancer tumors is accompanied by Treg infiltration (unpublished data), suggesting a correlation between IDO activity and Tregs in these tumors. However, the role of increased IDO expression in tumor cells in development of Treg cells is not clear. In this study, we investigated the potential effects of IDO on development of Treg cells in breast cancer tumors using a stable IDO-expressing Chinese hamster ovary (CHO) cell line.

## Materials and methods

### Cell lines and culture conditions

The Chinese hamster ovary (CHO) cell line was purchased from the Shanghai Institute of Cell Biology, Chinese Academy of Sciences (Shanghai, China). The breast cancer cell line MDA-MB-435s was obtained from American Type Culture Collection (Manassas, VA). Both cell lines were maintained in culture as adherent monolayer in RPMI-1640 (Gibco, Invitrogen Corp., Carlsbad, CA) medium supplemented with 10% fetal bovine serum (FBS), L-glutamine (1%) and penicillin (0.1%). Cells were incubated at 37°C in a humidified atmosphere with 5% CO2.

### Construction of a recombinant plasmid containing human IDO cDNA

Total RNA was isolated from breast cancer MDA-MB-435s cells using Trizol (Invitrogen, Carlsbad, CA) according to the manufacturer's instructions. A 1225 kb fragment encompassing the entire coding region of human IDO cDNA was obtained using RT-PCR (Takara, Dalian, China) with the following primer pair: sense 5'-AGATCTGCCACCATGGCACACGCTATGGAAAAC-3', and antisense 5'-GTCGACTTAACCTTCCTTCAAAAGGGATTTC-3'. The PCR products were inserted into the pMD19-T Simple Vector (Takara) using TA-cloning procedures, and sequencing analysis was used to identify the product of interest (pMD19-IDO).

### Establishment of stable transformants

For construction of stable transformants, pMD19-IDO and pIRES2-EGFP (Clontech, Santa Clara, CA) were digested with *Bgl*II and *Sal*I. The fragments of interests were recovered by agarose gel analysis, purified and ligated using T4 DNA ligase to generate the expression plasmid pIRES2-EGFP-IDO. The recombinant expression plasmid was confirmed by digestion with *Bgl*II and *Sal*I and sequencing. CHO cells were cultured in RPMI medium 1640 with 10% FBS for 24 h and then transfected with 10 μg of pIRES2-EGFP-IDO using a standard electroporation method (field strength of 350 V/cm, 60 μs, 1 pulse). The pIRES2-EGFP vector was used as a plasmid control, and CHO cells transfected with pIRES2-EGFP (CHO/EGFP) were used as a control cell line. The CHO/EGFP cells were established as described previously [11]. G418 (1 mg/ml) was added to the medium 48 h after transfection, and the medium was changed every 48 h for 4 weeks to obtain G418-resistant transformants. CHO cells containing pIRES2-EGFP-IDO were then identified by flow cytometric analysis.

### Detection of IDO gene transcripts in CHO cells and Foxp3 in co-cultured cells by RT-PCR

To investigate IDO gene integration into CHO cells, total RNA was isolated from CHO cells transfected with pIRES2-EGFP-IDO using Trizol. RT-PCR primers were: IDO (188 bp), sense 5'-CATCTGCAAATCGTGACTAAG-3'; antisense 5'-CAGTCGACACATTAACCTTCCTTC-3'. β-actin (186 bp) was used as an internal control; sense 5'-TGGCACCCAGCACAATGAA-3'; antisense 5'-CTAAGTCATAGTCCGCCTAGAAGCA-3'. cDNA was prepared by Oligo-(dT)15 from 1 μg of total RNA, and PCR was performed using a RT-PCR kit (Takara) according to the manufacturer's instructions. To analyze Foxp3 gene expression in co-cultured cells, total RNA was isolated using Trizol as described above, with Foxp3 (488 bp) primers, forward primer 5'-CCCACTTACAGGCACTCCTC-3'; reverse primer 5'-CTTCTCCTTCTCCAGCACCA-3'. RT-PCR was performed in a volume of 20 μL using 50 ng of RNA, 2 μL of 10× PCR buffer (Takara, Japan), 10 mM of each deoxynucleoside triphosphate (dNTP), 1 μL of each primer, 0.5 μL of Takara Taq polymerase and 13.5 μL of water. Conditions were 94° for 5 min, followed by 30 cycles of 30 s at 94°C, 30 s at 60°C, and 1 min at 72°C, with a final extension cycle of 72°C for 10 min. PCR products were analyzed by separation on 2% agarose gels.

### Quantitative real-time RT-PCR detection of Foxp3

Foxp3 gene expressions in T cells from different co-cultures were also assessed by quantitative real-time RT-PCR using β-actin mRNA as an internal control. Foxp3 primers, sense 5'-CCCACTTACAGGCACTCCTC-3'; antisense 5'-CTTCTCCTTCTCCAGCACCA-3'; β-actin, sense 5'-TGGCACCCAGCACAATGAA-3'; antisense 5'-CTAAGTCATAGTCCGCCTAGAAGCA-3'. PCR amplifications were performed in a 20 μl volume with each reaction containing 2 μl of 10× buffer, 0.4 μl (10 mmol/l) dNTP mixture, 1 μl (10 μmol/l) of each primer, 2 μl cDNA, 1 μl (20×) SYBR Green I, 3.2 μl (25 mmol/l) MgCl2, 1 U *Taq *DNA polymerase, 2.0 μl (1 mg/ml) BSA and 6.4 μl ddH2O. The thermal cycling conditions used were 95°C for 5 min, 94°C for 20 s, 60°C for 30 s, 72°C for 20 s, 80°C for 1 s; this was repeated for 40 cycles. All samples were measured in duplicate, and the average value was quantitated. To correct for sample-to-sample variation, an endogenous control, β-actin, was amplified with the target and served as an internal reference to normalize the data. The expression levels of Foxp3 relative to that ofβ-actin were calculated by using the 2-ddCt method.

### Western blot analysis

Total cellular extracts for Western blot analysis were obtained by lysis of 1 × 107 positively cloned CHO cells in lysis buffer (Pierce Biochemical, Rockford, IL), and the protein concentration was quantitated using the Micro BCA protein assay kit (Pierce). The extracts were heat denatured for 10 min in a 100°C water bath. Aliquots of cell lysates containing 50 μg of proteins were separated on a 12% SDS-polyacrylamide gel and transferred to PVDF membranes (Pall Corporation, Ann Arbor, MI). The filters were blocked with TBST buffer containing 2% BSA and incubated with an IDO monoclonal antibody (Chemicon International, Temecula, CA, 1:1000) overnight. Horseradish peroxidase-linked anti-mouse IgG (Chemicon, 1:5000) was then added, followed by immersion in SuperSignal West Pico Chemiluminescent Substrate (Pierce Biotechnology, Rockford, IL) for visualization of bands. The intensity of each band was recorded using the ChemiDoc XRS imaging system and was analyzed using Quantity One software (Bio-rad Laboratories, Milan, Italy). For detection of Foxp3 in co-cultures of IDO+ and CD3+ T cells (using mouse monoclonal antibody to Foxp3 [Clone PCH101, 1:1000 dilution; eBioscience]), inadherent cells were obtained 7 days after co-culture of CHO+ and CD3+ T cells, and the analysis was performed as described above.

### IDO activity assay

IDO expressing or untransfected (control) CHO cells (1 × 107) were incubated in RPMI 1640 with 10% FBS (Gibco). The supernatants of cell culture were harvested 72 h after incubation, and 2 mls were added to 0.1 g sulfosalicylic acid, followed by centrifugation at 4°C for 30 min. The concentrations of the enzymatic products were measured using the Hitachi amino acid L-8800-automatic analyzer (Hitachi, Tokyo, Japan). Enzyme activity was expressed as the product content per hour per milligram of protein.

### Co-culture of IDO+ CHO cells and CD3+T cells

Mononuclear cells were isolated from the peripheral blood of breast cancer patients using the CS-3000 Plus Blood Cell Separator (Baxter, Munich, Germany) according to the operator's manual. CD3+T cells were isolated and purified using the RosetteSep Human CD3 Depletion Cocktail kit (StemCell Technologies Inc., Vancouver, BC, Canada) according to the manufacturer's instructions. Informed consent was obtained from all subjects, and the study was approved by the University Ethics Committee. CHO/EGFP cells or CHO cells with stable IDO expression (1 × 105) were seeded per well of a 24-well plate, and 2 × 106 purified CD3+T cells and 200 U/ml human recombinant IL-2 (R&D Systems) were added. The cells were incubated in RPMI 1640 medium with 10% FBS at 37°C in a 5% CO2 incubator. The medium was changed every 2-3 days for 7 days. We added 1-MT, the specific inhibitor of IDO at concentration of 1 mM in the co-culture system composing of CHO/IDO cells and CD3+T cells to elucidate the regulatory effect of IDO both in promoting apoptosis and increasing Tregs.

### Flow cytometry assay

Co-cultured cells were harvested after 96 h for analysis of apoptosis. The apoptosis levels of T cells in the harvested cells (1 × 106/ml), which were gated using PE-Cy5 labeled anti-CD3 monoclonal antibody, were assessed by FITC labeled Annexin V and PI (BD Pharmingen, San Diego, CA) staining. As a positive control for apoptosis, CD3+ T cell apoptosis was also assessed 96 h after incubation in medium supplemented with 200 U/ml IL-2. To detect the proportion of Tregs after 7 days of co-culture, cells were harvested and incubated with 10 μl anti-CD4-PE-Cy5, 10 μl anti-CD25-FITC and 3 μl anti-CD127-PE (BD Pharmingen) at 4°C for 30 min in the dark. A minimum of 1 × 104 cells were washed 2 times with PBS and resuspended in 2% paraformaldehyde. Flow cytometric analysis was performed using a FACSAria flow cytometer (Becton Dickinson). The ratio of Tregs to CD3+T cells before culture was also assessed. The data were analyzed using Cell Quest software (Becton Dickinson).

### Statistical Analysis

All data were expressed as ($\bar{x}$ ± *SD*) and analyzed with statistical package SPSS 11.5 for Windows (SPSS Inc., Chicago, IL). The SNK-*q *method was used to determine statistically significant differences among the groups. One-way analysis of variance (ANOVA) and the Student's t test were used to determine the means of two different groups. *P *< 0.05 was considered statistically significant.

## Results

### Identification of the recombinant plasmid pIRES2-EGFP-IDO

**Figure 1:**
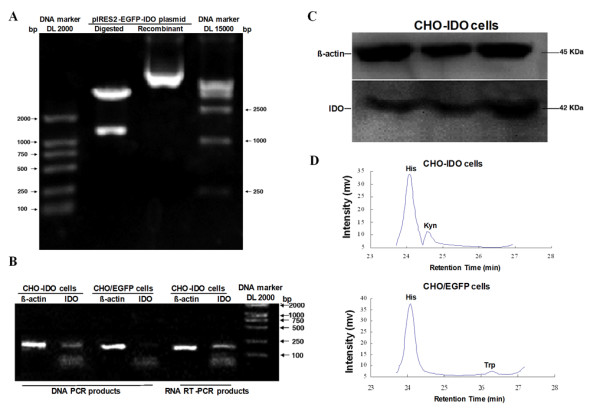
**Identification of IDO transfected CHO cells**. (A) Identification of recombinant plasmid pIRES2-EGFP-IDO by restriction enzyme analysis. The plasmid pIRES2-EGFP-IDO can be digested with *Bgl*IIand *Sal*I. xperiments in this figure and following figures were performed at least three times on separate occasions. (B) IDO gene integration and transcription by PCR and RT-PCR. (C) Western blot analysis of IDO protein expression in CHO-IDO cells using anti-IDO antibody. In transfected group, CHO cells transfected with IDO expressed the 42 kDa IDO protein, indicating that CHO cells stably transfected with IDO could produce IDO protein. (D) Analysis of free amino acids in culture supernatant. Amino acid level in CHO cells 72 h after IDO transfection: (His) 33.75 mg/L, (Kyn) 7.03 mg/L, (Trp) < 3 pmol. Amino acid level in CHO cells with *p*IRES2-EGFP transfection 72 h after culturing: (His) 38.12 mg/L, (Trp) 5.63 mg/L, (Kyn) < 3 pmol. His: histidine; Trp: trytophan; Kyn: kynurenine.

Digestion of the pIRES2-EGFP-IDO construct with *Bgl*II and *Sal*I liberated an IDO insert of the expected length (1225 kb), indicating that the plasmid was successfully constructed (Figure 1A). Analysis of IDO expression by PCR using genomic DNA, or by RT-PCR using total RNA, yielded a 188 bp fragment; meanwhile, no IDO expression was detected in CHO/EGFP cells, indicating that we could specifically detect the integration into the CHO cell genome and transcription of the transfected IDO gene (Figure 1B). Western blot analysis showed that the stably transfected IDO+ CHO cells expressed the 42 kDa IDO protein (Figure 1C). Kynurenine (8.14 ± 1.02 mg/L) but not tryptophan (< 3 pmol) was detected in the culture supernatant 72 h after the CHO cells were incubated with the IDO construct. However, tryptophan (5.85 ± 0.74 mg/L) but not kynurenine was detected in the culture supernatant of CHO/EGFP cells, indicating that IDO expressed by transfected CHO cells possessed functional activity and could metabolize tryptophan (Figure 1D).

### Effect of IDO+ CHO cells on CD3+T cell apoptosis

**Figure 2:**
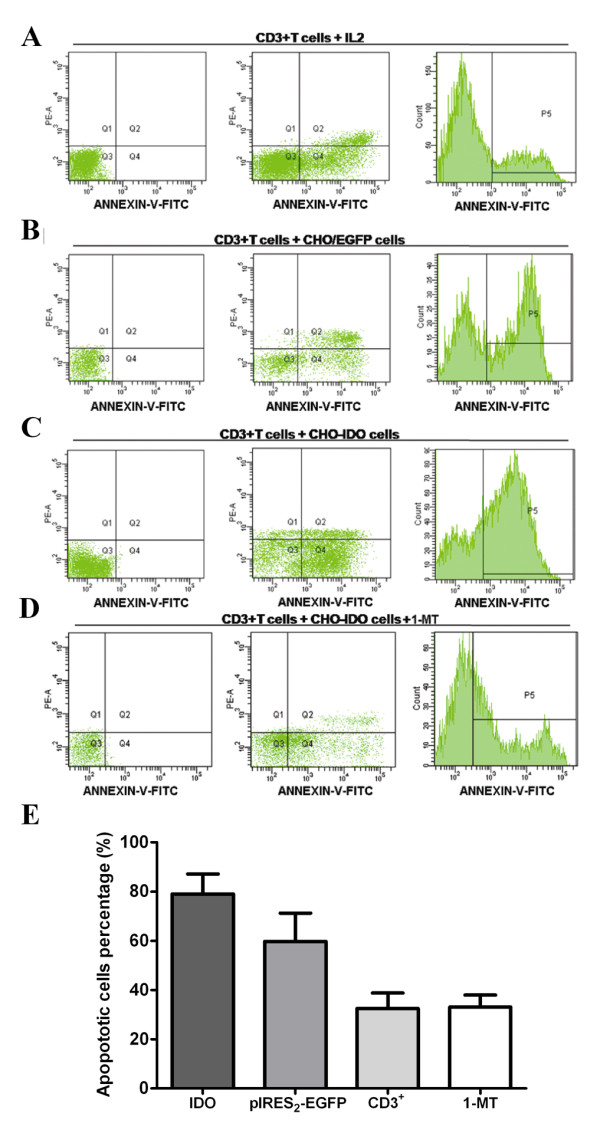
**Effect of IDO^+ ^CHO cells on CD3^+^T cell apoptosis**. (A) Representative FACS scatter plots of CD3^+^T cells apoptosis 72 h after culture with 200 U/ml human recombinant IL-2. (B) Representative FACS scatter plots of CD3^+^T cells apoptosis 72 h after co-culture with CHO/EGFP cells. (C) Representative FACS scatter plots of apoptotic CD3^+^T cells 72 h after co-culture with CHO cells transfected with IDO. (D) Representative FACS scatter plots of apoptotic CD3^+^T cells 72 h after co-culture with CHO cells transfected with IDO and inhibitor 1-MT. (Q4 region represents cells in the early process of apoptosis; P5 represents the total population of apoptotic CD3+T cells) (E) Relative percentages of apoptotic cells (Annexin V positive and PI negative cells). The columns showed the average (%) ± SD from 3 independent experiments. The differences were statistically significant (*P *< 0.05), indicating that CHO cells with IDO transfection can significantly induce apoptosis in T cells.

After 72 h of co-culture of CD3+T cells and IDO+ CHO cells, 79.07 ± 8.13% of CD3+T cells were apoptotic compared with 59.80 ± 11.46% of CD3+ T cells co-cultured with CHO/EGFP cells, and 32.40 ± 6.40% of CD3+ T cells that were cultured alone. The differences were statistically significant (*P *< 0.05), indicating that IDO+ CHO cells could induce significant T cell apoptosis. Furthermore, after added the 1-MT, the specific inhibitor of IDO in co-culture of CD3+T cells and IDO+ CHO cells, the apoptosis could not be induced (only 33.1 ± 4.87% of CD3+T cells were apoptotic) (Figure 2).

### In vitro *induction of peripheral CD4 + CD25 + CD127- T cells by IDO+ CHO cells in the peripheral blood of breast cancer patients*

**Figure 3:**
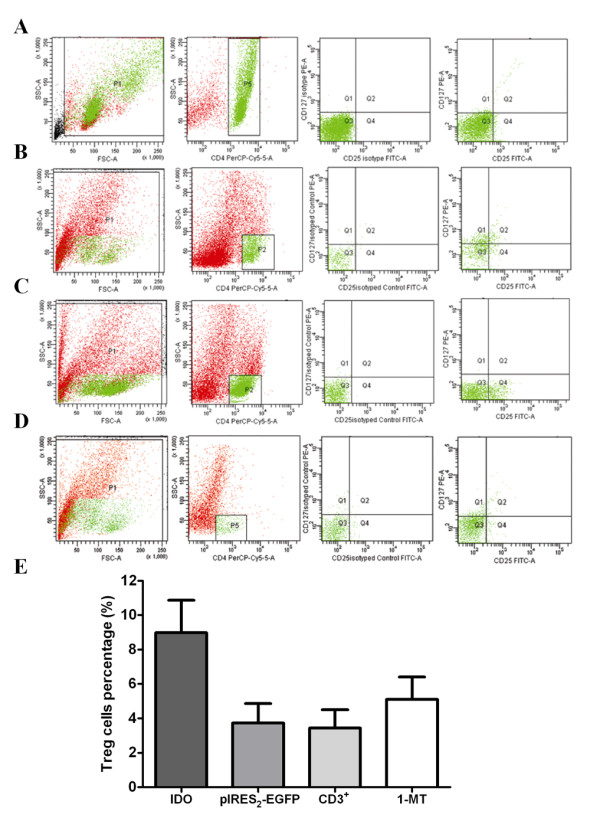
**Inductive effect of CHO cells with IDO transfection on Tregs**. (A) Representative FACS scatter plots of the CD4^+^CD25^+^CD127^- ^T cells in CD3^+ ^T cells 7 days after incubation. (B) Representative FACS scatter plots of CD4^+^CD25^+^CD127^- ^T cells 7 days after co-culture with CHO/EGFP cells. (C) Representative FACS scatter plots of CD_4_^+^CD_25_^+^CD_127_^- ^T cells 7 days after co-culture with IDO^+ ^CHO cells. (D) Representative FACS scatter plots of CD_4_^+^CD_25_^+^CD_127_^- ^T cells 7 days after co-culture with IDO^+ ^CHO cells and inhibitor 1-MT. (P2 region represents CD4^+ ^T cells, Q4 region represents CD4^+^CD25^+^CD127^- ^T cells.) (E) Relative percentages of CD4^+^CD25^+^CD127^- ^T cells in CD4^+ ^T cells. The columns showed the average (%) ± SD from 3 independent experiments. IDO^+ ^CHO cells had more Tregs in T cells after co-culture than in control groups. The differences were statistically significant (*P *< 0.05).

Mononuclear cells isolated from the peripheral blood of breast cancer patients were incubated with IDO+ CHO cells to assess the effect of IDO expression on Treg cells. After 7 days of incubation of 2 × 106 CD3+ T cells in media containing 200 U/ml IL-2, CD4+CD25+CD127- Tregs were 3.43 ± 1.07% of the CD3+T cell population. However, after 7 days of co-culture of 1 × 105 CHO cells expressing IDO or EGFP and 2 × 106 CD3+ T cells, CD4+CD25+CD127- Tregs were 8.98 ± 1.88% of the CD3+T cell population in co-cultures with IDO+ CHO cells, but were only 3.73 ± 1.12% of the CD3+T cell population in co-cultures with CHO/EGFP cells (Figure 3). The proportion of Tregs in co-cultures of CD3+ T cells and IDO+ CHO cells was higher than in the other two groups, and the differences were statistically significant (*P *< 0.05). After added the inhibitor 1-MT, CD4+CD25+CD127-Tregs were 5.1 ± 1.30% of the CD3+T cell population in co-cultures with IDO+ CHO cells. It confirmed that the IDO had the function to induce the peripheral Tregs.

### RT-PCR analysis of Foxp3 gene expression

**Figure 4:**
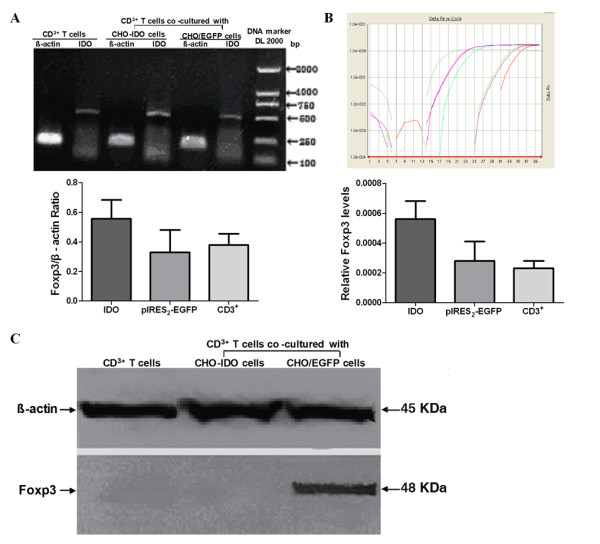
**Foxp3 expression in T cells after co-culture was detected by RT-PCR, Real-time PCR or Western blot**. (A) Analysis of RT-PCR products of Foxp3 and comparison of the gray scale value between Foxp3 and β-actin by agarose gel electrophoresis. Three separate experiments were carried out. RT-PCR product of β-actin and Foxp3 from the total mRNA isolated from CD3^+^T cells cultured with growth medium, or from the T cells co-cultured with IDO gene-transfected CHO cells, or from the T cells co-cultured with CHO/EGFP cells. The value of the Foxp3 and β-actin gray scale ratio in T cells after 7 days of co-culture with IDO gene-transfected CHO cells was higher than in the control groups (*P *< 0.05). (B) Expression of Foxp3 gene analyzed by real-time RT-PCR. Three separate experiments were carried out. Amplification curve of Foxp3 in the IDO gene-transfected group and the control groups. Expression of Foxp3 in T cells after 7 days of co-culture with IDO gene-transfected CHO cells was higher than that in the control groups (*P *< 0.05). (C) Expression of Foxp3 analyzed by Western blot analysis. Three separate experiments were carried out. Expression of Foxp3 protein in the CD3^+^T cells cultured with growth medium for 7 days; or 7 days after co-culture with CHO/EGFP cells; or 7 days after co-culture with IDO^+ ^CHO cells. No Foxp3 protein was detected in the control groups.

Seven days following co-culture of IDO+ CHO cells and CD3+ T cells, Foxp3 gene expression was detected in the CD3+ T cells by RT-PCR analysis. CD3+T cells alone and CD3+T cells co-cultured with CHO/EGFP cells were used as negative controls. The value of the Foxp3 and β-actin gray scale ratios in CD3+ T cells co-cultured with IDO+ CHO cells, CD3+ T cells and CD3+ T cells co-cultured with CHO/EGFP cells were 0.5567 ± 0.1271, 0.3283 ± 0.1530 and 0.3800 ± 0.0748, respectively. The value of the Foxp3 and β-actin gray scale ratio in the T cells co-cultured with IDO+ CHO cells was higher than in the control groups (*P *< 0.05) (Figure 4A).

### Quantitative real-time RT-PCR analysis of Foxp3 gene expression

Foxp3 gene expression was detected in CD3+T cells after 7 days of co-culture with IDO+ CHO cells by quantitative RT-PCR analysis. CD3+T cells and CD3+T cells co-cultured with CHO/EGFP cells were used as negative controls. The relative expression of Foxp3 in CD3+ T cells from IDO+ CHO cell co-cultures, in CD3+ T cells and in CD3+T cells from co-cultures with CHO/EGFP cells were 0.00056 ± 0.00012, 0.00028 ± 0.00013 and 0.00023 ± 0.00005, respectively. Relative Foxp3 gene expression was higher in T cells co-cultured with IDO+ CHO cells than in T cells from the control groups (*P *< 0.05) (Figure 4B).

### Western blot analysis of Foxp3 expression

Foxp3 protein expression was detected in CD3+ T cells 7 days after co-culture with IDO+ CHO cells. CD3+T cells and CD3+T cells co-cultured with CHO/EGFP cells were used as negative controls. Cell lysates from T cells isolated from co-cultures with IDO+ CHO cells contained a 48 kDa protein band reactive to a Foxp3-specific monoclonal antibody. This band was not present in cell lysates from T cells from the control group cultures (Figure 4C).

## Discussion

IDO is expressed in many human and animal tissues and cells as well as on the surface of human tumor cells. An in-depth analysis is needed to identify the specific mechanisms that underly the role of IDO in tumor immune tolerance. Recent studies have shown that acute myeloid leukemia (AML) cells that express IDO can transform CD4+CD25-T cells into CD4+CD25+T cells [12]. However further study is needed to elucidate the mechanism behind this transformation and the relationship between IDO and Treg cells in solid tumors [13-18]. In this study, we constructed a stable cell line expressing IDO and carried out preliminary *in vitro *analysis of the induction effect of IDO on Tregs isolated from the peripheral blood of patients with breast cancer.

IDO is expressed both in tissues of patients with breast cancer and in breast cancer cell lines [19,20]. In this study, during the preparation of the IDO gene expression vector, we identified IDO gene expression in the human breast cancer cell lines MDA-MB-231, MDA-MB-435S, MDA-MB-453, SK-Br-3, T47D, ZR-75-1 and normal breast cells HBL-60; the gene was highly expressed in MDA-MB-435S, T47D, MCF-7. We also detected IDO expression in patients with primary breast cancer and in lymph nodes draining the tumor; IDO expression in lymph node tissue was consistent with results previously reported in the literature [4,21,22]. Moreover, in our previous study, we found that the proportion of CD4+CD25+ regulatory T cells in the peripheral blood of patients with breast cancer was higher than that in the peripheral blood of patients with benign breast tumors and healthy volunteers; the proportion of CD4+CD25+Tcells was directly related to tumor size [23]. This phenomenon suggests that in patients with breast cancer, a mechanism may exist that can increase the proportion of Tregs. We also added 1-MT, the specific inhibitor of IDO in the co-culture system composing of CHO/IDO cells and CD3+T cells to elucidate the regulatory effect of IDO both in promoting apoptosis and increasing Tregs. It demonstrated that 1-MT could efficiently reversed enhancement of T cells apoptosis and increased Tregs proportion in vitro. It implied that IDO is indeed responsible for the changes observed in vitro.

Some studies have indicated a close relationship between IDO and regulatory T cells. Some dendritic cells in the lymph nodes draining tumors that express IDO had local infiltration of Tregs cells [21,22,22,24]. Furthermore, when IDO was expressed in the primary tumor of breast cancer patients, there was a direct correlation between an increase in volume of the primary breast cancer tumor and the proportion of Tregs in the peripheral circulation [23]. Tregs cells are also likely to be involved in IDO-mediated tumor immune tolerance [11,12]. To investigate this hypothesis, we established a CHO cell line that stably expressed IDO. Western blot analysis confirmed that CHO cells transfected with IDO expressed IDO protein with an expected molecular weight of approximately 42 kDa. At the same time, we detected a decrease in tryptophan in the culture medium, and an increase in its metabolite kynurenine, suggesting that IDO expressed by the transfected cells was functional and could lead to the depletion of tryptophan in the environment. Analysis of apoptosis after co-culture of IDO-expressing CHO cells and CD3+T cells isolated from the peripheral blood of patients with breast cancer showed that a significantly higher proportion of CD3+T cells were apoptotic than in the control group, suggesting that IDO may affect the T cell proliferation and induce T cell apoptosis. In our recent study, we found that cell proliferation and IL-2 synthesis triggered by the TCR activating anti-CD3 monoclonal antibody OKT3 was inhibited in T-cells which were co-cultured with IDO-expressing CHO cells. Furthermore, co-cultured of CHO/IDO with T-cells could inhibit Vav1 mRNA and protein expression in T-cells. However, an inhibitor of IDO, 1-MT, attenuated CHO/IDO-induced decrease of T-cell proliferation, IL-2 levels in T-cells and inhibition of Vav1 [11]. These data suggested that Vav1 is a target molecule involved in the regulatory effect of IDO on T-cells.

Whether IDO can induce the maturation and differentiation of Tregs is unclear. Investigation into the relationship between IDO expression and regulation of Tregs is likely to be key to revealing a tumor immune tolerance-related mechanism [11,25]. A recent experiment showed that in patients with acute myeloid leukemia, IDO-expressing tumor cells can induce the transformation of CD4+CD25-T cells to CD4+CD25+T cells [12]. In this study, we explored the inductive effect of IDO on Tregs isolated from the solid tumors of patients with breast cancer, and used low expression of CD127 as a more accurate and specific surface molecular marker of inhibitory Tregs [9,10]. We detected an increase in CD4+CD25+CD127- regulatory T cells in the CD3+T cell population from co-cultures of IDO-expressing CHO cells and CD3+T cells isolated from the peripheral blood of patients with breast cancer. This phenomenon may be due to the IDO induced differentiation of CD3+T into CD4+CD25+CD127- cells, but further study will be needed to confirm this conclusion.

## Conclusions

Endogenous IDO may be involved in a variety of peripheral tolerance mechanisms and immunosuppressive responses, as well as having a role in other cellular mechanisms. We established a cell line that stably expressed IDO and preliminarily confirmed that active expression of IDO could induce apoptosis in T cells isolated from the peripheral blood of patients with breast cancer; we confirm the role of IDO in the maturation and development of Tregs in breast cancer patients. This study provides an experimental basis for further study into the mechanism underlying the interaction between IDO and Tregs in tumor immunity.

## Competing interests

The authors declare that they have no competing interests.

## Authors' contributions

JS carried out the molecular genetic studies, participated in the sequence alignment and drafted the manuscript. JY carried out the immunoassays and drafted the manuscript. HL and LY participated in the sequence alignment. FW and WY performed the statistical analysis. JL and XR conceived of the study, and participated in its design and coordination. All authors read and approved the final manuscript.
